# UK COVID-19 lockdown: 100 days of air pollution reduction?

**DOI:** 10.1007/s11869-020-00937-0

**Published:** 2020-09-11

**Authors:** J.E. Higham, C. Acosta Ramírez, M.A. Green, A. P. Morse

**Affiliations:** School of Environmental Sciences, University of Liverpool, Roxby Building, Liverpool, UK

**Keywords:** COVID-19, Air quality, Monitoring, Public health

## Abstract

On the 23 March 2020, a country-wide COVID-19 lockdown was imposed on the UK. The following 100 days saw anthropogenic movements quickly halt, before slowly easing back to a “new” normality. In this short communication, we use data from official UK air-quality sensors (DEFRA AURN) and the UK Met Office stations to show how lockdown measures affected air quality in the UK. We compare the 100 days post-lockdown (23 March to 30 June 2020) with the same period from the previous 7 years. We find, as shown in numerous studies of other countries, the nitrogen oxides levels across the country dropped substantially (∼ 50%). However, we also find the ozone levels increased (∼ 10%), and the levels of sulphur dioxide more than doubled across the country. These changes, driven by a complex balance in the air chemistry near the surface, may reflect the influence of low humidity as suggested by Met Office data, and potentially, the reduction of nitrogen oxides and their interactions with multiple pollutants.

## Introduction

The global pandemic emerging from a novel strain of severe acute respiratory syndrome coronavirus 2 (SARS-CoV-2; COVID-19) has ravaged the world in 2020. This infectious disease has shown to have a significant effect on mortality especially in vulnerable groups such as the elderly, those with cardiovascular disease, diabetes, respiratory diseases and cancer (Williamson et al. 2020). On the 23 March 2020, the United Kingdom (UK) government imposed a country-wide lockdown, closing businesses and urging people to stay at home. As a result anthropogenic movements, i.e. motor vehicle usage radically decreased. The UK Department for Transport (DfT) reported on the day of lockdown there had already been a reduction in motor vehicle transport to 69% of normal and in the following days, this reached a low of 23% (13 April 2020) before steadily increasing back to 77% 100 days after the lockdown (30 June 2020). During these 100 days, motor vehicle usage was reduced on average to 52% of normal (Government U 2020). In several studies carried out in different countries, it was found reducing motor vehicle usage drastically decreased the amounts of nitrogen oxides and particulate matter, generally having a positive effect on air quality (Collivignarelli et al. 2020; Muhammad et al. 2020; Dantas et al. 2020; Tobías et al. 2020; Mahato et al. 2020; Singh and Chauhan 2020; Chauhan and Singh 2020; Bashir et al. 2020). These findings were likely to be mirrored in the UK. According to Public Health England, transport in the UK accounts for half of the nitrogen oxides (NO_*x*_) and substantial amounts of particulate matter with a diameter less than 2.5 microns (PM2.5), sulphur dioxide (SO_2_) and volatile organic compounds (VOCs), as shown in Table 1. A review of pollutant exposure shows that a reduction in NO_*x*_, SO_2_, particulate matter and VOCs presents potential benefits to human health. In contrast, high concentrations of NO_*x*_ increase mortality (Chen et al. 2007; Esplugues et al. 2011; Maheswaran et al. 2012; Hesterberg et al. 2009; PERSHAGEN et al. 1995), SO_2_ causes respiratory problems (Pikhart et al. 2001; Tertre et al. 2002; Martins et al. 2002), particulate matter has been linked to diabetes and cognitive disorders (Pope and Dockery 2006; Santibañez et al. 2013; Xing et al. 2019) and VOCs are also detrimental to health (Miekisch et al. 2004). Among the factors influencing the balance of these pollutants are meteorological conditions, the atmosphere oxidative capacity and human and natural sources of emissions. Changes in air chemistry composition can lead to a non-linear series of chemical and physical transformations (Atkinson 2000; He et al. 2014).

**Table 1:** Summary of percentage of UK air pollutants created by motor vehicles

	Non-road transport	Road-transport
NO_*x*_ (%)	16.8	33.6
SO_2_ (%)	8.3	0.7
PM2.5 (%)	3.6	12.4
VOC (%)	1.6	4.9

In the UK, land surface pollutant observations are recorded by the Department for Environment, Food and Rural Affairs (DEFRA) Automatic Urban and Rural Network (AURN), with a network of ∼ 300 hourly measuring sensors. Additionally, the UK Meteorological Office (Met Office) has a network of ∼ 200 hourly measuring meteorological stations distributed across the country as part of the Met Office Integrated Data System (MIDAS). AURN measurements meet the required European Standards as set out in the European Ambient Air Quality Directive (2008/50/EC), measuring a combination of nitrogen dioxide (NO_2_), ozone (O_3_), SO_2_ and particulate matter of diameter 2.5 and 10 microns (PM2.5 and PM10). The Met Office stations measure all number of meteorological quantities including temperature, relative humidity (RH) and wind speed.

This short communication is structured as follows: First, we investigate the general changes of mass concentrations of NO_*x*_, SO_2_, PM2.5 and O_3_ on the whole of the UK during the lockdown period in comparison with the previous 7 years. Second, we analyse the regional effects considering major UK cities. Finally, we present a set of conclusions.

## Results and discussion

### UK-wide effects of lockdown

**Fig. 1:**
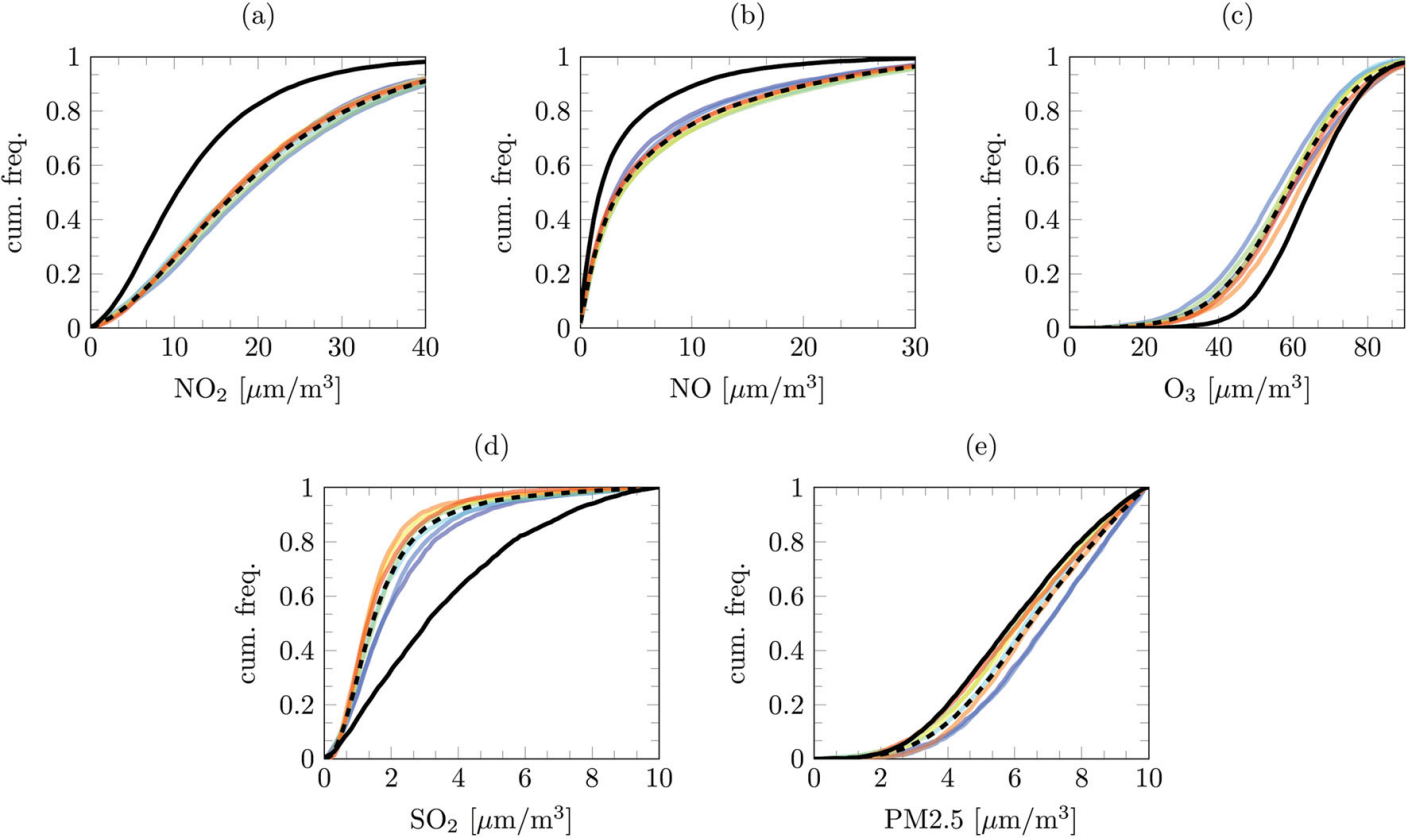
Cumulative distribution functions of all UK DEFRA AURN pollutant hourly readings recorded over 100 days post 23 March 2020 lockdown with comparisons of same period in previous 7 years. **a** NO_2_. **b** NO. **c** O_3_. **d** SO_2_. **e** PM2.5

**Fig. 2:**
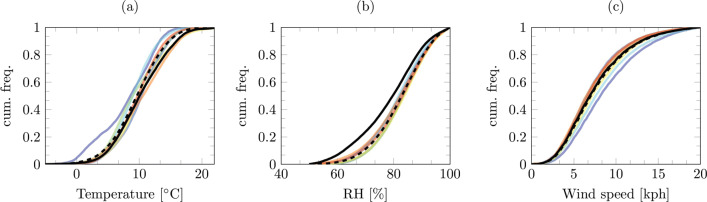
Cumulative distribution functions of all UK Met Office average hourly readings taken over 100 days post 23 March 2020 lockdown with comparisons of same period in previous 7 years. **a** Temperature. **b** Relative humidity. **c** Average wind speed

To investigate the effects of the lockdown on the whole country we create simple statistics from all of the sensors data. Figures 1 and 2 show cumulative distribution functions for the AURN and Met Office stations. These data are created from all of the stations at hourly intervals for the 100 days succeeding the lockdown. Using the same period (considering leap years), we compare the lockdown period with the previous 7 years. The summarised mean data are presented in Table 2.

**Table 2:** UK average DEFRA pollution & MIDAS meteorological readings 100 days following lockdown, compared with with 2019 and 7-year average

	2013	2014	2015	2016	2017	2018	2019	Av. 2013–2019	2020
SO_2_ (*μ* m/m^3^)	2.88	2.57	2.49	2.22	1.92	1.81	1.90	2.26	3.95
O_3_ (*μ* m/m^3^)	60.44	55.87	58.42	58.17	58.89	62.72	61.13	59.38	66.03
PM2.5 (*μ* m/m^3^)	12.48	13.29	9.36	9.88	9.83	11.45	11.90	11.17	9.14
NO (*μ* m/m^3^)	11.47	12.87	12.96	14.35	12.08	11.19	10.34	12.18	4.66
NO_2_ (*μ* m/m^3^)	22.80	24.72	22.79	24.18	21.90	22.42	21.64	22.92	13.21
Temp (°C)	8.22	10.50	9.21	9.53	10.67	10.75	9.96	9.83	10.56
RH (%)	81.88	83.27	81.72	83.42	83.38	81.66	82.55	82.55	78.60
Wind (kph)	9.77	7.73	8.90	7.98	8.55	7.81	7.85	8.37	8.30

From these summarised data, it is evident that the reduction in motor vehicles and anthropogenic movement had a substantial impact on air quality. However, it is of worthy consideration that, from the 7-year period, 2020 showed the highest temperatures and lower RH values (Fig. 2). From the cumulative distribution functions, it is notorious that, during the first 100 days of lockdown, there have been greater occurrences of higher temperature and lower relative humidity events. On average, during this period, the UK has been 1 °C hotter with ∼ 4% less relative humidity (in comparison with the 7-year average). Analogous to other studies (Collivignarelli et al. 2020; Muhammad et al. 2020; Dantas et al. 2020; Tobías et al. 2020; Mahato et al. 2020), the data show there has been a huge reduction in the concentration of NO_*x*_. UK-wide nitric oxide (NO) is reduced by ∼ 55% from 2019 and 61% from the 7-year average. Nitrogen dioxide (NO_2_) has also been reduced by 39% compared with 2019 and 42% the average of the previous 7 years. We present the components of nitrogen oxides separate (NO_*x*_ = NO + NO_2_) as several studies show NO_2_, NO and O_3_ are indistinguishably linked. The work of the Photochemical Oxidants Review Group (PORG) PORG (1997), Wang et al. (2019), and Leighton (2012) suggest that due to the complex atmospheric balance of air pollutants, a change in the ratio between the two nitrogen oxides would modify the ozone production. The summarised statistics validate this hypothesis: the larger decrease in NO_*x*_ should increase the O_3_ concentration. Similar to the finding in Milan (Collivignarelli et al. 2020), the summarised AURN data show, compared with both 2019 and the 7-year average, there is a ∼ 10% increase in O_3_ concentration. Although, as shown by Leighton (2012), the relationship is non-trivial, the O_3_ production is not just a function of NO_*x*_ but also meteorological conditions; the unseasonably warm weather during the lockdown could have further exacerbated the O_3_ production (Naja and Lal 2002). Ras et al. (2009) show this relationship is also further complicated by the presence of VOCs, which act as a precursor in ozone production; however, these data recorded by the AURN stations. Reducing NO_*x*_ is, of course, beneficial to human health, although evidence shows increased O_3_ levels may exacerbate respiratory issues (Jaffe et al. 2003) a factor likely to enhance the probability of complications relating to COVID-19.

From the summarised data (Table 2), it appears in the first 100 days of lockdown there has been a substantial increase in the concentrations of SO_2_. Compared with both 2019 and the 7-year average, SO_2_ levels have approximately doubled. The cumulative distribution functions show this higher average relates to an increase in higher concentration events. Typically sulphur dioxide in the UK has been created by industry; however, since the 1970s, it has seen a sharp decline. This increase in SO_2_ is unlikely to have been produced by point sources. One explanation for this increment could follow the findings of He et al. (2014), who show higher concentrations of NO_*x*_ promote the conversion of SO_2_ into sulphates. The ∼ 25% reduction in particulate matter (from both 2019 and the 7-year average) further support this explanation. Another explanation could follow the work of Cox and Penkett (1972) who relate a sulphur dioxide increase to relative humidity reduction and (Brimblecombe 1978) who shows a wet surface reduction (less rain) removes a SO_2_ sink. In contradiction to the previous statement, excess SO_2_ could be emanating from by point sources; Qian et al. (2007) show excess cremations in China increased sulphur dioxide concentration. One thing is clear the wind is unlikely to have a role in, the summarised Met Office data show there are changes in average UK wind speed compared with 2019 or the previous 7 years. The most likely explanation is a combination of all these findings. Undoubtedly, an increase in SO_2_ is not favourable for human health. Increased concentrations of SO_2_ are associated with dyspnea (shortness of breath) and cough (Pikhart et al. 2001) even short-term exposure to high concentrations can lead to hospitalisation of the elderly and vulnerable (Martins et al. 2002).

### UK regional effects

**Fig. 3:**
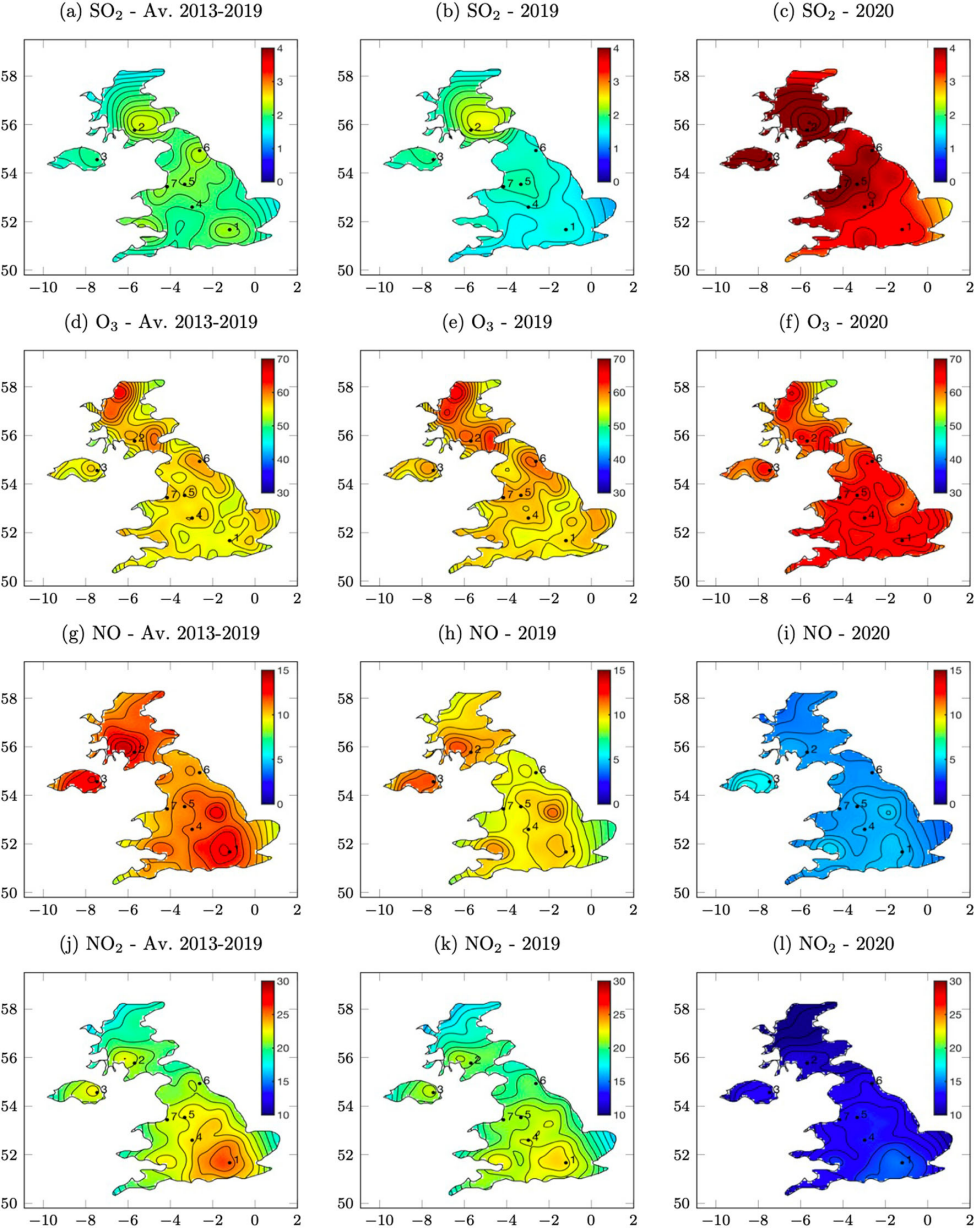
Contour plots created from UK AURN monitors showing average of the 100 days following lockdown and comparisons with 2019 and an average of the previous 7 years

To further elucidate the localised effects of lockdown on air-quality, we focus on seven large UK cities: London, Glasgow, Belfast, Birmingham, Manchester and Liverpool. In Fig. 3, we present contours of NO_2_, NO, O_3_ and SO_2_ and summarise their mean values at key UK cities in Table 3. These figures are created by interpolating using a multi-resolution, cubic, gridded-interpolator (Higham and Brevis 2019) and any outliers removed using the PODDEM alogrithm (Higham et al. 2016).

**Table 3:** UK largest cities average AURN pollution & Met Office meteorological readings in the 100 days following lockdown, compared with 2019 and 7-year average

City	Year	SO_2_ (*μ* m/m^3^)	O_3_ (*μ* m/m^3^)	PM2.5 (*μ* m/m^3^)	NO (*μ* m/m^3^)	NO_2_ (*μ* m/m^3^)	Temp (°C)	RH (%)
[1] London	2019	1.6 (+ 116%)	56.6 (+ 16%)	12.5 (− 26%)	10.1 (− 54%)	23.0 (− 36%)	7.9 (+ 21%)	81.3 (− 8%)
	Av. 2013–2019	1.9 (+ 82%)	48.4 (+ 35%)	10.2 (− 9%)	11.5 (− 59%)	22.4 (− 35%)	7.0 (+ 38%)	71.0 (+ 5%)
	2020	3.5	65.4	9.3	4.7	14.7	9.6	74.4
[2] Glasgow	2019	2.3 (+ 117%)	60.4 (+ 5%)	9.4 (− 25%)	11.0 (− 62%)	20.6 (− 44%)	5.9 (+ 18%)	83.8 (− 4%)
	Av. 2013–2019	2.0 (+ 152%)	50.3 (+ 25%)	8.0 (− 12%)	11.3 (− 63%)	18.7 (− 39%)	5.3 (+ 32%)	73.4 (+ 9%)
	2020	5.1	63.1	7.1	4.1	11.5	7.0	80.2
[3] Belfast	2019	1.9 (+ 168%)	58.2 (+ 8%)	10.4 (− 30%)	11.8 (− 54%)	21.6 (− 41%)	6.7 (+ 23%)	85.2 (− 9%)
	Av. 2013–2019	1.7 (+ 206%)	49.2 (+ 28%)	8.4 (− 14%)	11.4 (− 52%)	19.7 (− 36%)	5.7 (+ 45%)	75.1 (+ 3%)
	2020	5.1	62.9	7.3	5.5	12.7	8.3	77.2
[4] Birmingham	2019	1.6 (+ 130%)	57.3 (+ 16%)	12.0 (− 28%)	9.7 (− 54%)	21.9 (− 39%)	8.0 (+ 19%)	82.1 (− 8%)
	Av. 2013–2019	1.7 (+ 117%)	49.6 (+ 34%)	9.7 (− 10%)	10.0 (− 56%)	20.2 (− 34%)	7.1 (+ 34%)	72.0 (+ 5%)
	2020	3.7	66.2	8.7	4.4	13.4	9.5	75.7
[5] Manchester	2019	1.9 (+ 116%)	58.0 (+ 12%)	11.6 (− 28%)	9.6 (− 55%)	21.0 (− 37%)	7.0 (+ 18%)	82.7 (− 6%)
	Av. 2013–2019	1.9 (+ 114%)	49.3 (+ 32%)	9.3 (− 10%)	9.8 (− 56%)	19.5 (− 32%)	6.3 (+ 30%)	72.4 (+ 7%)
	2020	4.0	64.9	8.4	4.3	13.2	8.2	77.8
[6] Newcastle	2019	1.7 (+ 135%)	60.0 (+ 7%)	11.1 (− 29%)	9.0 (− 55%)	20.1 (− 39%)	6.4 (+ 18%)	82.2 (− 5%)
	Av. 2013–2019	2.0 (+ 98%)	50.8 (+ 26%)	8.9 (− 12%)	9.3 (− 57%)	18.5 (− 33%)	5.8 (+ 30%)	71.8 (+ 9%)
	2020	3.9	64.1	7.9	4.0	12.3	7.5	78.2
[7] Liverpool	2019	1.8 (+ 142%)	58.9 (+ 12%)	11.4 (− 28%)	9.2 (− 55%)	20.9 (− 38%)	6.8 (+ 19%)	79.6 (− 6%)
	Av. 2013–2019	1.9 (+ 130%)	50.2 (+ 32%)	9.3 (− 11%)	9.4 (− 56%)	19.1 (− 32%)	6.0 (+ 33%)	70.3 (+ 7%)
	2020	4.3	66.2	8.3	4.2	13.0	8.0	75.0
Av. UK	2019	1.9 (+ 108%)	61.1 (+ 8%)	11.9 (− 23%)	10.3 (− 55%)	21.6 (− 39%)	10.0 (+ 6%)	82.6 (− 5%)
	Av. 2013–2019	2.3 (+ 75%)	59.4 (+ 11%)	11.2 (− 18%)	12.2 (− 62%)	22.9 (− 42%)	9.8 (+ 7%)	82.6 (− 5%)
	2020	4.0	66.0	9.1	4.7	13.2	10.6	78.6

Bracketed percentage numbers relate to increase or decrease of quantities. Numbers before city names relate to city locations in contour plots

As expected, the contour plots comparing the 100 days following lockdown with 2019 and 7-year average show the NO_*x*_ high concentrations are situated around the densely populated cities. For all of the chosen cities, in the first 100 days of the lockdown, NO_2_ levels in all of the cities, except London and Glasgow, reduced in-line with the UK 2019 average (39%) with a 37–41% reduction. In comparison with 2019, the reduction in NO_2_ is slightly less (36%) but it is greater in Glasgow (44%). Compared with the 7-year average, all of the cities show a similar reduction in NO_2_ (32–36%) with Glasgow slightly higher (39%). All of these values are less than the UK’s 7-year average (42%), likely explained by the year on year decline of nitrogen dioxide emissions (see Table 1). The NO emissions follow a similar trend, compared with 2019, all cities apart from Glasgow reduced the NO production by 54–55%, in line with the UK average (55%). Glasgow had a more substantial reduction compared with 2019 (63%). Compared with the 7-year average in all of the chosen cities, the NO production ranges between 56 and 59% in contrast to the UK average of (62%), with a deeper reduction in Glasgow (63%). As expected for all of the sites, there is an increase in ozone. Following PORG (1997), Wang et al. (2019), and Leighton (2012), it would be expected the most sizeable increase in O_3_ would be in London, where there is a substantial reduction in the ratio of NO oxide to NO_2_ decrease, and vice versa for Glasgow. This expectation is partly confirmed by the data; compared with 2019 Glasgow had the smallest increase in O_3_ (5%), and London one of the greatest (16%) with other cities also recording similar increases ranging from 7 to 16% comparable with the UK average (8%). Notably, compared with 2019, Belfast produced only a small increase in ozone (8%). Compared with 2019, the increase in O_3_ concentrations, with exception to Belfast, correlated with the decrease in relative humidity at each location. Quite clearly the meteorological conditions are playing a role, on closer inspection, the change in relative humidity may not have had such an effect in Belfast as in 2019 their recorded relative humidity was higher than in the other cities. These findings also correlate with the comparison of O_3_ records with the 7-year average, London is the principal O_3_ producer and Glasgow the least. However, as demonstrated by the contour plots, the distribution of O_3_ across the UK is normally quite complex. As discussed by Sillman (1999), these non-linear complexities of ozone concentrations do extend into rural areas and in some cases, there can be higher concentrations in lesser populated areas. Although the contour plots of O_3_ demonstrate that whilst background O_3_ has also decreased, in cities, it has been far greater.

The data show a decrease in relative humidity relates to an increase in SO_2_. Records of Belfast show an increase of 168% in SO_2_ with a decrease in 9% relative humidity, in comparison with 2019. In Liverpool, Newcastle and Birmingham, SO_2_ emissions increased by 142%, 135% and 130% with anti-correlating reductions in relative humidity (5%, 6% and 8%). As with other pollutants, Glasgow has the smallest increase in SO_2_ (117%), and this also relates to the only slight change in relative humidity (4%). Despite London and Manchester having similar records, these cities do not follow the aforementioned trend both seeing an increase of 116% concentration of SO_2_ with a relative humidity decrease of 8% and 6%. However, the comparison with the 7-year average demonstrates the complexity of SO_2_ concentrations across the UK. By comparing 2019 with the 7-year average, it is notorious that in some cities (London, Birmingham, Manchester, Liverpool and Newcastle), there have been sizeable decreases in SO_2_ levels; however, in Belfast and Glasgow, these have been relatively small. It is also found that, compared with 2019, there is a positive correlation between the concentration of PM2.5 and SO_2_. As Belfast has the largest concentration increase of SO_2_, it also has the highest increase in PM2.5 (30% in 2019 and 24% over a 7-year average). Similarly, Glasgow also records the smallest increase in SO_2_ (25% and 23%). The complexities in the SO_2_ distribution across the UK are demonstrated in the contour plots. Being one of the most densely populated cities in the world, it could be assumed that London would have the highest concentrations of SO_2_, although, as the data show, more northern cities both in 2019 and in the 7-year average have higher concentrations. This is likely to be related to manufacturing and local sources, but also the meteorological conditions and higher relative humidity play a part in this distribution.

## Conclusions

Other COVID-19 air-quality studies of different countries have reported lockdowns and reduced anthropogenic movement has been excellent for air quality. The majority of these studies have mainly focused on the reductions in NO_*x*_ and VOCs; as in our findings, some have even shown mild increases in O_3_. However, to our knowledge, there have been no studies which have to investigate SO_2_. In this short communication, we find UK-wide SO_2_ levels are more than double in comparison with the previous 7 years. We show meteorological conditions as a potential factor for this increase, but the study of other factors is necessary. One relevant aspect could be point-source pollution, although most likely this increase is based on an imbalance in the complex air chemistry caused by the removal of NO_*x*_.

The reduction in NO_*x*_ is beneficial to human health, although an increase in SO_2_ and its associated effects on human health may outweigh these gains. Most worryingly we also find, during the 100 days following lockdown, the levels of SO_2_ were far worse in the Northern regions of the UK. These regions typically have higher rates of deprivation and unemployment, and areas more susceptible to the COVID-19 virus, but this discussion is far beyond the scope of the present study. In conclusion, from this brief communication, it is important to note that the complex and relatively stable air composition in the surface layer can be disrupted in a short period of time by the drastic reduction of primary emissions from anthropogenic sources. For the case of UK, getting cleaner air from a drastic NO_*x*_ reduction may not be as straightforward as it seems.
